# Description and DNA barcoding of *Crematogaster fraxatrix* Forel, 1911 and two new closely related species from Cambodia and Indonesia (Hymenoptera, Formicidae)

**DOI:** 10.3897/zookeys.374.5874

**Published:** 2014-01-29

**Authors:** Shingo Hosoishi, Kazuo Ogata

**Affiliations:** 1 Institute of Tropical Agriculture, Kyushu University, 6-10-1 Hakozaki, Higashi-ku, Fukuoka 812-8581 Japan

**Keywords:** *Crematogaster fraxatrix*, taxonomy, new species, lectotype designation, DNA barcoding, cytochrome *c* oxidase I

## Abstract

*Crematogaster fraxatrix* Forel, 1911 and two new species, *C. chhangi* **sp. n.** and *C. simboloni* **sp. n.**, are described from Cambodia and Indonesia, respectively. DNA sequences were generated for *C. fraxarix* and the two newly described species using 3 amplications of two regions of the mitochondrial gene COI with a total of 1129 bp. The mean interspecific divergences are 9.4% and 23.5% for *C. fraxatrix* vs. *C. chhangi*, *C. simboloni*, respectively. DNA sequences reveal that *C. simboloni* is found to be genetically distinct from the other two species, but *C. chhangi* is not distinct from *C. fraxatrix*.

## Introduction

*Crematogaster fraxatrix* was described by Forel (1911) based on the worker specimens from Malaysia (Borneo). This species is presently assigned to the subgenus *Crematogaster* (Blaimer 2012b). A recent molecular work re-classified the former sixteen subgenera into two larger subgenera, *Crematogaster* and *Orthocrema* (Blaimer 2012b). The subgenus *Crematogaster* is the largest, including more than 220 species, its workers generally have anteriorly broader petiole (‘flared’-shape in Blaimer 2012b), but *Crematogaster ferrarii*, *Crematogaster fraxatrix* and *Crematogaster ransonneti* uniquely have the petiole broader in the middle portion among Asian *Crematogaster* fauna. The close relationship between *Crematogaster ferrarii* and *Crematogaster fraxatrix* was also suggested by a molecular phylogeny (Blaimer 2012c). However, *Crematogaster fraxatrix* can be easily distinguished by the densely sculptured mesopleuron from *Crematogaster ferrarii*, by the weakly concave metanotal groove from *Crematogaster ransonneti*, respectively. In the course of our recent examination of *Crematogaster* specimens collected from southeast Asia, two distinct species related to *Crematogaster fraxatrix* were found, which are here described as new species. Cytochrome oxidase I (COI) sequence data from *Crematogaster fraxatrix* was further compared with that of the two new species. DNA barcodes have been recently used in biodiversity studies of ant species (Smith et al. 2005), and are used as an additional and powerful method in integrative taxonomy (Schlick-Stiner et al. 2010). They can thus provide a useful reference for the identification of Asian *Crematogaster* species. Our analysis included not only in the conventional 5’ DNA barcoding region, but also the 3’ region of COI region. The relationship between *Crematogaster fraxatrix* and the two new species is discussed, based on morphological features and sequence divergence.

## Materials and methods

### Sources of material and abbreviations

Specimens were examined and/or deposited in the collections listed below. Codes for public institutions mainly follow those in Brandão (2000). Nest series samples, most of which were recently collected, are represented as colony codes, e.g., “SH12-Cam-70.”

BMNH The Natural History Museum, London, U.K.

CASC California Academy of Sciences, San Francisco, CA, USA.

FRIM Forest Research Institute Malaysia, Kepong, 52109 Kuala Lumpur, Malaysia.

KUM Kyushu University, Fukuoka, Japan.

MCZC Museum of Comparative Zoology, Harvard University, Cambridge, MA, USA.

MHNG Musee d’Histoire Naturelle, Geneva, Switzerland.

MZB Museum Zoologicum Bogoriense, Cibinong, Java, Indonesia.

NHMB Naturhistorisches Museum, Basel, Switzerland.

THNHM Thailand Natural History Museum, Technopolis, Khlong Luang, Pathum Thani, Thailand.

### Measurements and indices

Most observations were made using an Olympus SZX12 microscope. Images were taken using a Canon EOS 50D with a Canon MP-E 65 mm 1-5 × macro lens, then processed using Combine ZM. Measurements were made with an Olympus SZX12 stereomicroscope using micrometers. All measurements are expressed in millimeters, recorded to the second decimal place. The measurements for petiole and postpetiole follow Longino (2003).

Head Width (HW): Maximum width of head in full-face view, excluding the eyes.

Head Length (HL): Perpendicular distance from vertex margin to line tangent anteriormost projections of clypeus in full-face view.

Cephalic Index (CI): HW/HL × 100.

Scape Length (SL): Length of the first antennal segment, excluding the neck and basal condyle.

Scape Index (SI): SL/HW × 100.

Eye Length (EL): Maximum length of the compound eye.

Pronotal Width (PW): Maximum width of the pronotum in dorsal view.

Weber’s Length of the mesosoma (WL): Diagonal length, measured in lateral view from the anterior margin of the pronotum (excluding the collar) to the posterior extremity of the propodeal lobe.

Propodeal Spine Length (PSL): measured from tip of propodeal spine to closest point on outer rim of propodeal spiracle.

Petiole Length (PtL): Length of the petiole in lateral view (see Longino 2003, fig. 2).

Petiole Width (PtW): Maximum width of petiole in dorsal view.

Petiole Height (PtH): Height of the petiole in lateral view (see Longino 2003, fig. 2).

Postpetiole Length (PpL): Length of the postpetiole in lateral view (see Longino 2003, fig. 2).

Postpetiole Width (PpW): Maximum width of postpetiole in dorsal view, excluding the helcium.

Petiole Height Index (PtHI): PtH/PtL × 100.

Petiole Width Index (PtWI): PtW/PtL × 100.

Postpetiole Width Index (PpWI): PpW/PpL × 100.

Waist Index (WI): PpW/PtW × 100.

### Genetic analysis

Genomic DNA was extracted from tissues rich in mitochondria (e.g. legs) using a DNeasy Blood & Tissue kit (Qiagen, Maryland, USA). A 497 bp region of the mitochondrial genome, including barcoding regions of the cytochrome oxidase I (COI) was amplified via the polymerase chain reaction (PCR) using the following combinations of the primers, “LepF1” 5’-ATTCAACCAATCATAAAGATATTGG-3’ and “C_ANTMR1D-RonIIdeg_R” 5’-GGRGGRTARAYAGTTCATCCWGTWCC-3’ (used only for PCR), and “MLepF1” 5’-GCTTTCCCACGAATAAATAATA-3’ and “LepR1” 5’-TAAACTTCTGGATGTCCAAAAAATCA-3’ (Hebert et al. 2004; Fisher and Smith 2008; Hajibabaei et al. 2006). Reactions were carried out at 10 µl volumes in a PCR Thermal Cycler MP (TaKaRa Bio Inc.) under the following conditions: a first cycle of 94 °C for 2 min, followed by 5 cycles of 94 °C for 40 sec, annealing at 45 °C for 40 sec, and 72 °C for 1 min, then 36 cycles of 94 °C for 40 sec, annealing at 51 °C for 40 sec, and finally 72 °C for 1 min for the COI. A 632 bp region of the 3’ region of COI was amplified via the polymerase chain reaction (PCR) using primers “Jerry” 5’-CAACATTTATTTTGATTTTTTGG-3’ and “Pat” 5’-TCCAATGCACTAATCTGCCATATTA-3’ (Simon et al. 1994). Reactions were carried out at 10 µl volumes in a PCR Thermal Cycler MP (TaKaRa Bio Inc.) under the following conditions: a first cycle of 94 °C for 1 min, followed by 5 cycles of 94 °C for 1 min, annealing at 48 °C for 90 s, and 72 °C for 90 s, then 30 cycles of 94 °C for 1 min, annealing at 51 °C for 90 s, and finally 72 °C for 90 s for the COI.

PCR products were visualized on a 1% agarose E-Gel 96-well system (Invitrogen), and then purified with 1.0 µl of ExoSAP-IT (GE Healthcare Life Sciences). All products were sequenced in both directions (except for C_ANTMR1D-RonIIdeg_R) using BigDye Terminator v3.1 (Applied Biosystems) on an ABI 3100 Avant DNA Sequencer (Applied Biosystems) at the Faculty of Science, Kyushu University, Fukuoka. Some fragments were removed prior to alignment, due to low quality. After trimming, the 5’ DNA barcoding region sequenced in this study were 497bp, therefore these sequences were not strictly DNA barcodes. Using the three primer sets, non-overlapping fragments of 244, 253 and 632 bp were sequenced respectively. DNA sequence data for eight individuals of three *Crematogaster* species were thus generated, and deposited at DNA Data Base of Japan, DDBJ (with accession numbers shown in Table 1). All data were registered in the project called: “Crematogaster ants in Asia” (CREAA) on Barcode of Life Database (BOLD). Among the three species, *Crematogaster chhangi* was sequenced from one nest series (SH12-Cam-70) from Cambodia, and *Crematogaster simboloni* was successfully sequenced only from one Krakatau specimen. Contigs were assembled using Vector NTI Advance TM ver. 11 (Invitrogen Corp.) and subsequently aligned by eye. Genetic distances were estimated using the Kimura-2-parameter (Kimura 1980) distances with MEGA 5 (Tamura et al. 2011). The phylogenetic tree was estimated using Neighbor-Joining (NJ) (Saitou and Nei 1987) in the program MEGA 5.

**Table 1.** Specimen data and DDBJ accessions.

Species	Voucher specimen	Locality	DDBJ accession numbers	
			first half of COI	second half of COI
*Crematogaster chhangi*	KUMANT001	Cambodia, Koh Kong	AB828274, AB828377	AB828264
*Crematogaster fraxatrix*	KUMANT002	Malaysia, Peninsula	AB828275, AB828381	AB828265
*Crematogaster fraxatrix*	KUMANT003	Malaysia, Borneo	AB828276, AB828382	AB828266
*Crematogaster fraxatrix*	KUMANT004	Malaysia, Borneo	AB828277, AB828383	AB828267
*Crematogaster fraxatrix*	KUMANT005	Malaysia, Borneo	AB828278, AB828384	AB828268
*Crematogaster fraxatrix*	KUMANT006	S. Thailand	AB828279, AB828385	AB828269
*Crematogaster fraxatrix*	KUMANT007	Malaysia, Peninsula	AB828280, AB828378	AB828270
*Crematogaster simboloni*	KUMANT008	Indonesia, Krakatau	AB828281, AB828386	AB828271
*Crematogaster osakensis*	KUMANT009	Japan	AB828282, AB828379	AB828272
*Crematogaster modiglianii*	KUMANT010	Malaysia, Peninsula	AB828283, AB828380	AB828273

## Results

Intraspecific variation for *Crematogaster fraxatrix* was 4.19% on average, with a range of 0.4–6.9%. Relatively large divergence in *Crematogaster fraxatrix* (6.2–6.9%) was recorded when comparing Peninsular to Bornean specimens. By contrast, the divergence within Peninsular (0.7–1.2%) or Bornean specimens (0.4–0.9%) was low. Interspecific sequence divergence was 17.1% on average, and ranged from 8.1–24.6% (Table 2). The lowest interspecific genetic distance occurred between specimens of *Crematogaster chhangi* and Bornean *Crematogaster fraxatrix* (8.1%).

**Table 2.** Percent mitochondrial cytochrome c oxidase I (COI) sequence divergence among species of *Crematogaster chhangi*, *Crematogaster fraxatrix* and *Crematogaster simboloni*.

Species	*Crematogaster chhangi* [Cambodia]	*Crematogaster fraxatrix* [M. Peninsula]	*Crematogaster fraxatrix* [Borneo]	*Crematogaster fraxatrix* [Borneo]	*Crematogaster fraxatrix* [Borneo]	*Crematogaster fraxatrix* [S. Thailand]	*Crematogaster fraxatrix* [M. Peninsula]
*Crematogaster chhangi* [Cambodia]							
*Crematogaster fraxatrix* [M. Peninsula]	0.105						
*Crematogaster fraxatrix* [Borneo]	0.082	0.069					
*Crematogaster fraxatrix* [Borneo]	0.082	0.067	0.009				
*Crematogaster fraxatrix* [Borneo]	0.081	0.067	0.004	0.007			
*Crematogaster fraxatrix* [S. Thailand]	0.108	0.007	0.065	0.063	0.063		
*Crematogaster fraxatrix* [M. Peninsula]	0.107	0.013	0.063	0.062	0.062	0.008	
*Crematogaster simboloni* [Krakatau]	0.243	0.241	0.227	0.23	0.226	0.246	0.241

The neighbor-joining tree (Fig. 9) shows *Crematogaster chhangi* sister to *Crematogaster fraxatrix*, with high bootstrap support (100%). Among the three species examined, *Crematogaster simboloni* was distinctly separated from the *Crematogaster chhangi* and *Crematogaster simboloni* with higher genetic divergence: 24.3% to *Crematogaster chhangi* and 22.6% to 24.6% to *Crematogaster fraxatrix*. *Crematogaster chhangi* is distinguished from *Crematogaster fraxatrix* only in having an acutely developed subpetiolar process, whereas *Crematogaster simboloni* is quite different from *Crematogaster chhangi* and *Crematogaster fraxatrix* in having a densely sculptured promesonotum.

### Key to species of *Crematogaster fraxatrix*-group

**Table:** 

1	Promesonotum sculptured	*Crematogaster simboloni*
–	Promesonotum not sculptured, but feebly striated with longitudinal rugulae	2
2	Propodeal spiracles strongly flattened dorsoventrally. Subpetiolar process acutely developed	*Crematogaster chhangi*
–	Propodeal spiracles oval in shape. Subpetiolar process weakly developed	*Crematogaster fraxatrix*

## Taxonomy

### Crematogaster (Crematogaster) chhangi sp. n.

http://zoobank.org/DDB2C024-BAE9-4A7B-9006-1C4BA647F716

http://species-id.net/wiki/Crematogaster_chhangi

Figs 1–2

#### Type locality.

CAMBODIA: Koh Kong, 11°31'N, 103°09'E, 19.v.2012, S. Hosoishi. (SH12-Cam-70).

#### Type-specimens.

**Holotype** worker: pinned. Original label: CAMBODIA, Koh Kong, 11°31'N, 103°09'E, 19.v.2012, S. Hosoishi leg., SH12-Cam-70, arboreal; deposited at THNHM.

Eight paratype workers: pinned, same data as holotype; deposited at BMNH, CASC, FRIM, KUM, MCZC, MHNG, MZB, NHMB.

#### Measurements and indices.

HW 0.64–0.83; HL 0.64–0.75; CI 100–111; SL 0.57–0.61; SI 73–86; EL 0.13–0.16; PW 0.37–0.42; WL 0.7–0.78; PSL 0.13–0.18; PtL 0.17–0.21; PtW 0.16–0.19; PtH 0.13–0.15; PpL 0.11–0.12; PpW 0.14–0.18; PtHI 65–82; PtWI 89–100; PpWI 127–150; WI 82–95 (holotype and eight paratype workers measured).

#### Diagnosis.

This species is similar to *Crematogaster fraxatrix*, but can be distinguished by the dorso-ventrally flattened propodeal spiracles and acutely developed subpetiolar process in the worker caste. The COI divergence between *Crematogaster chhangi* and *Crematogaster fraxatrix* did not seem relatively high (8.1–10.8% K2P distances) (cf. Blaimer 2012a), but the two species are clearly separated from each other by the characters shown above.

#### Worker description.

Workers presumably monomorphic. Posterior corners of head rounded. Anterior clypeal margin slightly concave in the median portion. Compound eyes not projecting beyond lateral margins of head in full face view. Scape reaching posterior corner of head. Antennal club 3-segmented. Pronotal dorsum with distinct ridges laterally. Mesonotal dorsum with lateral ridges. Mesonotum not higher than pronotum in lateral view; forming same dorsal outline with pronotum in lateral view. Metanotal groove straight in dorsal view, deep and forming a concave region between mesonotum and propodeum. Propodeal spiracles oval, flattened dorso-ventrally, located on the lateral sides of propodeum; the horizontal diameter more than two times larger than the vertical diameter. Propodeal spines developed long, directed upward and straight. Petiole broader in the middle portion. Subpetiolar process acutely developed. Postpetiole weakly bilobed, but without longitudinal median sulcus. Petiole slightly wider than postpetiole in dorsal view. Erect pilosity sparse. Scape with abundant erect to suberect setae. Dorsal face of head with suberect setae. Clypeus with suberect setae; one pair of longer setae directed medially on anteriormost portion. Anterior clypeal margin with one single setae and one pair of longer setae, mixed with some shorter setae on the sides. Mesosoma with sparse erect setae. Fourth abdominal tergite with erect to suberect sparse setae. Dorsal surface of head generally smooth and shining, but feeble rugulae between frontal carinae; longitudinal rugulae surrounding antennal sockets and on gena. Clypeus weakly striated with longitudinal rugulae. Promesonotum striated with feeble rugulae. Lateral surface of pronotum smooth and shining. Mesopleuron sculptured. Lateral surface of propodeum generally smooth, but with feeble rugulae on the lower portion. Body color brown.

**Figures 1–2.**
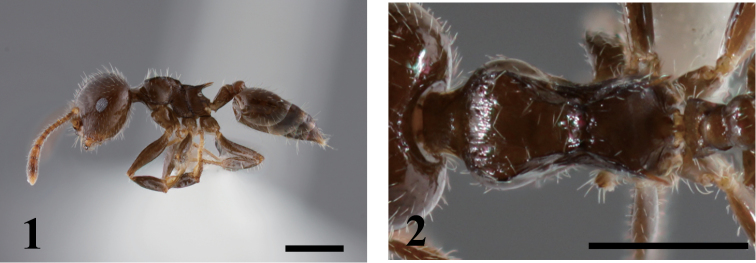
*Crematogaster chhangi*. **1** lateral view **2** dorsal view of mesosoma.

#### Distribution.

This species is known only from the type locality of Cambodia.

#### Etymology.

This species is dedicated to Mr. Phourin Chhang, Forestry Administration of Cambodia, who helped with field surveys in Cambodia.

### Crematogaster (Crematogaster) fraxatrix

Forel

http://species-id.net/wiki/Crematogaster_fraxatrix

Figs 3–6

Crematogaster fraxatrix Forel, 1911: 28 Worker syntypes from MALAYSIA: Sarawak, Borneo (*Haviland*) [MHNG, NHMB, examined]. Combination in *Crematogaster (Acrocoelia)*: Emery 1922: 151; in *Crematogaster (Crematogaster)*: Bolton 1995: 166; Blaimer 2012b: 55.

#### Type material examined.

MALAYSIA: Sarawak, Borneo (Haviland). Lectotype worker by present designation: top specimen of three specimens of one pin.

#### Other material examined.

THAILAND: 5 workers, Khlong Klai Stn., Khao Nan N. P., Nakhon S. Thamarat, 13.iii.2007 (TH07-SKY-22) (Sk. Yamane); MALAYSIA: 2 workers, Ulu Gombak, Selangor, 09.iii.2009 (SH09-Mal-51) (S. Hosoishi); 2 workers, Mt. Ophir, Gunung Ledan, Johor, 11.x.2011 (SH11-Mal-47) (S. Hosoishi); 3 workers, Lambir Hill’s National Park, Borneo, 21-27.ii.2009 (Y. Hashimoto).

#### Measurements and indices.

HW 0.7–0.98; HL 0.64–0.93; CI 105–114; SL 0.58–0.68; SI 69–91; EL 0.13–0.18; PW 0.37–0.62; WL 0.69–0.95; PSL 0.13–0.21; PtL 0.18–0.25; PtW 0.18–0.25; PtH 0.13–0.17; PpL 0.11–0.15; PpW 0.16–0.23; PtHI 65–74; PtWI 94–111; PpWI 123–155; WI 83–92 (thirteen workers measured).

#### Diagnosis.

This species is similar to *Crematogaster chhangi*, but can be distinguished by the oval-shaped propodeal spiracles and weakly developed subpetiolar process in the worker caste. Based on COI divergence, the specimens from Peninsular Malaysia were separated from the Bornean specimens with a high support value (Fig. 9). This is presumably due to lack of gene flow between the populations, but they showed no distinct morphological differences between each other. The COI divergence of 0-9.3% (K2P distances) was recorded within *Crematogaster ranavalonae* clade in Madagascar (Blaimer 2012a). Further geographic sampling is therefore needed to determine whether the variation of 0.4–6.9% (K2P distances) represents the intraspecific variation or includes some interspecific variation.

#### Worker description.

Workers with weak polymorphism in size. Posterior corners of head rounded in smaller worker, but squared in larger workers. Anterior clypeal margin slightly concave in the median portion. Compound eyes not projecting beyond lateral margins of head in full face view. Scape reaching posterior corner of head. Antennal club 3-segmented. Pronotal dorsum with distinct ridges laterally. Mesonotal dorsum with lateral ridges. Mesonotum slightly higher than pronotum in larger workers in lateral view. Metanotal groove straight in dorsal view, deep and forming a concave region between mesonotum and propodeum. Propodeal spiracles dorso-ventrally oval, located on lateral sides of propodeum; the horizontal diameter slightly larger than the vertical diameter even in smaller workers. Propodeal spines long, directed upward and straight. Petiole broader in the middle portion. Subpetiolar process developed as small, blunt denticle. Postpetiole weakly bilobed, but without longitudinal median sulcus. Petiole slightly wider than postpetiole in dorsal view.

Sparsely hirsute with erect setae. Scape with abundant erect to suberect setae. Dorsal face of head with suberect setae. Clypeus with suberect setae; one pair of longer setae directed medially on anteriormost portion. Anterior clypeal margin with one single setae and one pair of longer setae, mixed with some shorter setae on the sides. Mesosoma with sparse erect setae. Fourth abdominal tergite with sparse erect to suberect setae.

Dorsal surface of head generally smooth and shining, but feeble rugulae between frontal carinae; longitudinal rugulae surrounding antennal sockets and on gena. Clypeus weakly striated with longitudinal rugulae. Pronotum striated with feeble rugulae. Mesonum weakly striated with feeble rugulae. Lateral surface of pronotum smooth and shining. Mesopleuron sculptured, but the central portion relatively smooth. Lateral surface of propodeum with feeble rugulae.

Body color reddish-brown to black.

**Figures 3–6.**
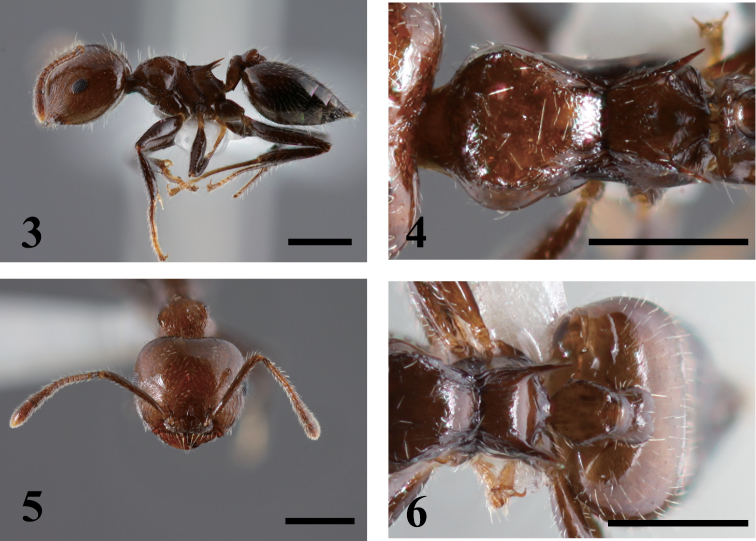
*Crematogaster fraxatrix*. **3** lateral view **4** dorsal view of mesosoma **5** full face view **6** dorsal view of petiole and postpetiole.

#### Distribution.

This species is known from southern Thailand and Malaysia (Peninsular and Borneo).

### Crematogaster (Crematogaster) simboloni sp. n.

http://zoobank.org/FFEDDB4D-EE5D-47D6-90A3-473F11F91056

http://species-id.net/wiki/Crematogaster_simboloni

Figs 7–8

#### Type locality.

INDONESIA: Rakata Island, Krakatau Islands, 06°09'S, 105°28'E, 11.x.2000, H. Simbolon.

#### Type-specimens.

**Holotype** worker: pinned. Original label: INDONESIA, Rakata Island, Krakatau Islands, 06°09'S, 105°28'E, 11.x.2000, H. Simbolon leg., deposited at MZB.

Eight paratype workers: pinned, same data as holotype; deposited at BMNH, CASC, FRIM, KUM, MCZC, MHNG, NHMB, THNHM.

#### Other material examined.

INDONESIA: 11 workers, Rakata Island, Krakatau Islands, 11.x.2000 (K. Ogata); 3 workers, Rakata Island, Krakatau Islands, 10.x.2000 (K. Ogata); 4 workers, Rakata Island, Krakatau Islands, 11.x.2000 (S. Matsui); 1 worker, Rakata Island, Krakatau Islands, 10.x.2000 (S. Matsui); 1 worker, Rakata Island, Krakatau Islands, 30.xii.2006 (Sk. Yamane); 1 worker, Rakata Island, Krakatau Islands, 31.xii.2006 (Sk. Yamane).

#### Measurements and indices.

HW 0.59–0.72; HL 0.57–0.67; CI 102–108; SL 0.49–0.55; SI 75–85; EL 0.11–0.15; PW 0.34–0.39; WL 0.62–0.74; PSL 0.1–0.12; PtL 0.16–0.2; PtW 0.16–0.19; PtH 0.12–0.15; PpL 0.11–0.12; PpW 0.15–0.17; PtHI 72–82; PtWI 95–113; PpWI 125–155; WI 83–100 (fourteen workers measured).

#### Diagnosis.

This species is similar to *Crematogaster chhangi* and *Crematogaster fraxatrix*, but can be easily distinguished from these by the sculptured promesonotum in the worker caste. The COI divergence between *Crematogaster simboloni* and *Crematogaster chhangi* (24.3% K2P distances), as well as *Crematogaster simboloni* and *Crematogaster fraxatrix* (22.6 to 24.6% K2P distances) were also high.

#### Worker description.

Workers monomorphic. Posterior corners of head rounded. Anterior clypeal margin slightly concave in the median portion. Compound eyes projecting slightly beyond lateral margins of head in full face view. Scape reaching posterior corner of head. Antennal club 3-segmented. Pronotal dorsum with distinct ridges laterally. Mesonotal dorsum with lateral ridges. Mesonotum slightly higher than pronotum in lateral view. Metanotal groove straight in dorsal view, deep and forming a concave region between mesonotum and propodeum. Propodeal spines long, directed upward and straight. Propodeal spiracles oval, flattened dorso-ventrally, located on the lateral sides of propodeum, or the postero-lateral corners; the horizontal diameter slightly larger than the vertical diameter. Petiole broader in the middle portion. Subpetiolar process undeveloped. Postpetiole weakly bilobed with feeble median sulcus. Petiole as wide as postpetiole in dorsal view. Sparsely hirsute with erect setae. Scape with abundant erect to suberect setae. Dorsal face of head with suberect setae. Clypeus with suberect setae; one pair of longer setae medially on anteriormost portion. Anterior clypeal margin with one single setae and one pair of longer setae, mixed with some shorter ones on the side. Mesosoma with short and sparse erect setae. Fourth abdominal tergite with few erect to suberect setae. Dorsal surface of head generally smooth and shining, but feeble rugulae between frontal carinae; longitudinal rugulae surrounding antennal sockets and on gena. Clypeus striated with longitudinal rugulae. Pronotum striated with longitudinal rugulae with the sculptured space; the longitudinal rugulae separated from anterior mesonotal margin. Mesonotum sculptured. Lateral surface of pronotum smooth and shining. Mesopleuron sculptured, but the central portion relatively smooth. Propodeal dorsum sculptured anteriorly. Lateral surface of propodeum weakly sculptured and striated with feeble rugulae. Body color brown.

**Figures 7–8.**
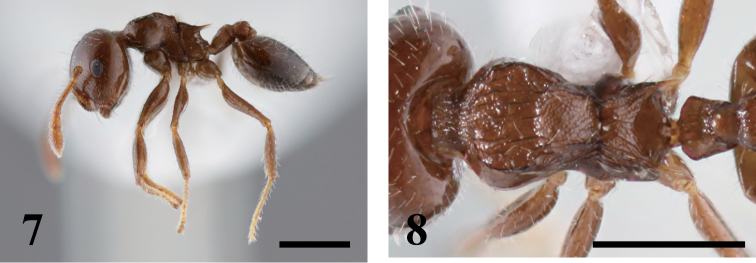
*Crematogaster simboloni*. **7** lateral view **8** dorsal view of mesosoma.

#### Distribution.

This species is known only from Indonesia (Krakatau).

#### Etymology.

This species is dedicated to Dr. Herwint Simbolon, Research Centre for Biology, Lembaga Ilmu Pengetahuan Indonesia (The Indonesian Institute of Sciences), who collected the type material.

**Figure 9.**
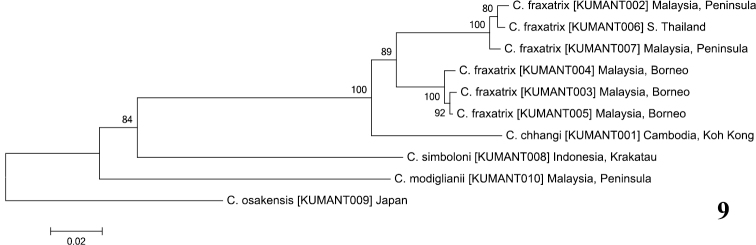
Neighbor-joining (Saitou and Nei 1987) tree of genetic distances (Kimura-2-parameter model (Kimura 1980), Bootstrap 1000 bootstrap replicates) of cytochrome *c* oxidase I (COI) for three *Crematogaster* species. Numbers on the nodes show the bootstrap values (>50%). Numbers in parentheses are specimen sample IDs.

## Supplementary Material

XML Treatment for Crematogaster (Crematogaster) chhangi

XML Treatment for Crematogaster (Crematogaster) fraxatrix

XML Treatment for Crematogaster (Crematogaster) simboloni
